# No Serological Evidence of Trachoma or Yaws Among Residents of Registered Camps and Makeshift Settlements in Cox’s Bazar, Bangladesh

**DOI:** 10.4269/ajtmh.21-0124

**Published:** 2021-05-03

**Authors:** Gretchen M. Cooley, Leora R. Feldstein, Sarah D. Bennett, Concepcion F. Estivariz, Lauren Weil, Rajendra Bohara, Maya Vandenent, ASM Mainul Hasan, Mohammad Saifuddin Akhtar, M. Salim Uzzaman, Mallick Masum Billah, Laura Conklin, Daniel C. Ehlman, Kingsley Asiedu, Anthony W. Solomon, ASM Alamgir, Meerjady Sabrina Flora, Diana L. Martin

**Affiliations:** 1 Division of Parasitic Diseases and Malaria, Centers for Disease Control and Prevention, Atlanta, Georgia;; 2 Epidemic Intelligence Service, Centers for Disease Control and Prevention, Atlanta, Georgia;; 3 Global Immunization Division, Centers for Disease Control and Prevention, Atlanta, Georgia;; 4 National Center for Immunization and Respiratory Diseases, Centers for Disease Control and Prevention, Atlanta, Georgia;; 5 World Health Organization, Dhaka, Bangladesh;; 6 United Nations Children’s Fund, Dhaka, Bangladesh;; 7 World Health Organization, Geneva, Switzerland;; 8 Institute of Epidemiology, Disease Control and Research, Dhaka, Bangladesh

## Abstract

Successful achievement of global targets for elimination of trachoma as a public health problem and eradication of yaws will require control efforts to reach marginalized populations, including refugees. Testing for serologic evidence of transmission of trachoma and yaws in residents of registered camps and a Makeshift Settlement in Cox’s Bazar District, Bangladesh, was added to a serosurvey for vaccine-preventable diseases (VPDs) conducted April–May 2018. The survey was primarily designed to estimate remaining immunity gaps for VPDs, including diphtheria, measles, rubella, and polio. Blood specimens from 1- to 14-year-olds from selected households were collected and tested for antibody responses against antigens from *Treponema pallidum* and *Chlamydia trachomatis* using a multiplex bead assay to evaluate for serologic evidence of the neglected tropical diseases (NTDs) yaws and trachoma, respectively. The prevalence of antibodies against two *C. trachomatis* antigens in children ranged from 1.4% to 1.5% for Pgp3 and 2.8% to 7.0% for CT694. The prevalence of antibody responses against both of two treponemal antigens (recombinant protein17 and treponemal membrane protein A) tested was 0% to 0.15% in two camps. The data are suggestive of very low or no transmission of trachoma and yaws, currently or previously, in children resident in these communities. This study illustrates how integrated serologic testing can provide needed data to help NTD programs prioritize limited resources.

## INTRODUCTION

Between late August 2017 and December 2017, more than 650,000 people moved into the Cox’s Bazar area in Bangladesh, joining approximately 300,000 others who had arrived during earlier waves of migration.^1^ The two preexisting registered camps, Kutupalong and Nayapara, and Makeshift Settlements expanded with the new influx. Many partners are working to address the health issues facing this densely populated and vulnerable population. In the first 7 weeks of 2018, large numbers of cases of acute watery diarrhea (*N* = 36,533) and acute respiratory infection (*N* = 74,034) were reported.^2^ Measles and diphtheria outbreaks were also reported.^3^

From September 2017 to March 2018, vaccination campaigns were implemented to reduce the risk of transmission of measles, diphtheria, and other vaccine-preventable diseases (VPDs).^4^ To guide further vaccination activities, a household vaccination coverage and serosurvey was undertaken in April–May 2018 that included integrated serological surveillance using multiplex bead assays for targets linked to parasitic and neglected tropical diseases (NTDs) that have elimination goals. The assay panel included antigens specific for *Chlamydia trachomatis* and *Treponema pallidum*; ocular strains of *C. trachomatis* cause trachoma, and *T. pallidum* subspecies *pertenue* causes yaws.

Trachoma is targeted for elimination as a public health problem.^5^ Districts with ≥ 5% prevalence of the sign trachomatous inflammation—follicular (TF) in 1- to 9-year-olds require interventions that include antibiotic mass drug administration to affected communities and efforts to address facial cleanliness and environmental improvement.^6^ Myanmar has eliminated trachoma (https://www.who.int/southeastasia/news/detail/11-09-2020-myanmar-eliminates-trachoma-who), and Bangladesh is not thought to require interventions.^7^ However, displaced populations deserve special consideration. The ongoing high incidence of acute watery diarrhea in these communities suggests overcrowding and poor access to sanitation, conditions also found in trachoma-endemic communities of other countries.

Myanmar was previously endemic for yaws, but like many countries that carried out truncated eradication campaigns in the mid-20th century, the current status of yaws in Myanmar is unknown.^8^ Bangladesh is not known to have ever been endemic for yaws.^8^ After the discovery that a single oral dose of azithromycin could effectively treat yaws,^9^ the WHO revived the goal of yaws eradication, aiming for complete interruption of transmission—the absence of new cases—globally by 2020.

Serologic testing for antibody responses against *C. trachomatis* antigens is gaining traction as a potential approach for conducting surveillance in areas that have achieved elimination criteria for trachoma and ceased interventions. Seroprevalence of anti–*C. trachomatis* antibodies typically increases with age among 1- to 9-year-olds in areas with ongoing transmission^10–12^ but remain relatively flat, with low seroconversion rates,^13^ in the absence of transmission^14–16^ and in settings where the presence of TF does not correlate with ocular *C. trachomatis* infection.^17,18^ Serologic testing is standard for yaws diagnosis: the *T. pallidum* particle agglutination (TPPA) assay reflects a history of *T. pallidum* infection, whereas nontreponemal tests such as rapid plasmin reagin (RPR) detect antibodies against host molecules released in response to infection, giving an indication of current or recent exposure. These tests together are diagnostic for active yaws or syphilis (caused by *T. pallidum* sp. *pallidum*; serologically indistinct from *T. pallidum pertenue*). We have recently adapted recombinant antigens from *T. pallidum* for use on the multiplex bead assay platform, with good correlation between responses to the antigen recombinant protein17 (rp17) and TPPA tests, and between responses to treponemal membrane protein A (TmpA) and RPR titers.^19^

In this study, we measured antibody responses to *C. trachomatis* and *T. pallidum* antigens to opportunistically evaluate serologic evidence of yaws and trachoma transmission, respectively, in the population in two settlements of Cox’s Bazar, Bangladesh. We used samples collected in an integrated serosurvey that had been primarily designed to estimate remaining immunity gaps for VPDs.

## METHODS

### Sampling.

A full description of the methods and sampling used during the integrated household coverage and serologic survey in April–May 2018 is published elsewhere.^4^ Representative samples of households in Nayapara Registered Camp, Kutupalong Registered Camp, and the Makeshift Settlements were selected. In Nayapara and Kutupalong, simple random sampling of households was conducted using household registration lists. In the Makeshift Settlements, a multistage cluster-sampling approach was undertaken.

In each selected household, one child aged 6 months to 6 years and one child aged 7 to 14 years were randomly selected for interview. Selected children aged 1 to 14 years were eligible for dried blood spot (DBS) specimen collection. Sample size estimates assumed 1) 65% of households would have at least one child aged 6 months to 6 years (60% for DBS specimen collection, given that age was further restricted to those aged 1–6 years) and 60% to 65% would have at least one child aged 7 to 14 years; 2) a household nonresponse rate (i.e., no one home) of 20% in the Registered Camps and 5% in the Makeshift Settlements; 3) a child nonresponse rate of 1% for all children for the survey component of the assessment; and 4) a 10% refusal rate for DBS specimen collection among children aged 1 to 6 years and 20% for children aged 7 to 14 years. Target enrollment was estimated assuming vaccination coverage with three doses of diphtheria-containing vaccine, and the proportion of children with protective antibody levels for tetanus was 50%.

An interview with an available caregiver was conducted in Rohingya in or near the home, using a standardized questionnaire that included questions about demographics, length of time in Registered Camps or Makeshift Settlements, vaccination history, and recent health concerns. The expected enrollment was 1,089 children aged 6 months to 6 years and 1,055 children aged 7 to 14 years. Because data collection in Nayapara took place during Ramadan and many children were expected to observe fasting, DBS samples were not collected from 7- to 14-year-olds there.

Enrollment in Kutupalong was stopped early because the camp leadership did not want the community to participate in the survey at that time.

From eligible children, we collected three to four drops of blood using a single finger prick per child (expected number of children to be enrolled for DBS sample collection: 914 aged 1–6 years and 787 aged 7–14 years). Blood was collected onto Whatman 903 Protein Saver Cards (GE Healthcare, Pittsburgh, PA). One large drop of blood was applied to each circle (≈70 µL/≈13 mm diameter), with up to four circles filled per child. The DBS were air-dried for at least 4 hours with protection from light, dust, and insects in plastic boxes inside dark back packs. After drying, DBS were placed in sealable plastic bags with a 5-g desiccant sachet and humidity indicator card, then stored in larger sealable plastic bags with additional desiccant and a humidity indicator card, which were checked daily and changed weekly. DBS were sent to CDC laboratories in the United States for testing using a multiplex bead assay to measure antibody levels against a panel of antigens from several VPDs and parasitic and neglected tropical diseases, including the antigens Pgp3 and CT694 from *C. trachomatis* and rp17 and TmpA from *T. pallidum*.

### Multiplex bead Aassay.

A 3-mm circular punch, assumed to have a 50% hematocrit, was taken from a blood-filled circle of each card and eluted in 125 µL Elution Buffer (1X phosphate buffered saline [PBS], 0.05% sodium azide, 0.3% Tween-20; Calbiochem, Burlington, MA) overnight at 4°C in a 96-well plate. Elutions were diluted to a final serum concentration of 1:400 in in Buffer B (1X PBS, 0.5% polyvinyl alcohol, 0.8% polyvinylpyrrolidone, 0.5% casein, all Sigma, Burlington MA; 0.3% Tween-20, 0.02% sodium azide, filtered with a 0.2 µm filter) containing 3 µg/mL of *Escherichia coli* extract and incubated overnight at 4°C.

Specimens were incubated in duplicate in a 96-well plate with beads, followed by incubation with anti-human immunoglobulin (Ig)G and IgG4 (both Southern Biotech, Birmingham AL) and streptavidin-linked R-phycoerythrin reporter (Invitrogen, Waltham, MA) [20]. Antibody binding was reported as median fluorescence intensity (MFI) using a MAGPIX instrument (Luminex, Austin, TX). Background from a DBS blank was subtracted from the (MFI-BG). Specimens having a coefficient of variation of > 15% between the MFI-BG of duplicate wells for any bead region were repeated.

Cutoffs for seropositivity were determined by receiver operator characteristic curves. For *C. trachomatis* antigens, the positive panel consisted of sera from 101 children from the United Republic of Tanzania previously classified as antibody-positive; the negative panel was derived from 74 U.S.-born 1- to 9-year-olds residing in New York who tested negative for *Chlamydia* spp. and antichlamydial antibodies using the microimmunofluorescence assay.^20^ The rp17 positive panel consisted of 39 samples from 4- to 15-year-olds from yaws-endemic areas who were positive by TPPA, and the negative panel consisted of 20 samples from individuals living in yaws-endemic areas who had tested negative by both TPPA and RPR, plus 74 samples from U.S.-born and -resident 1- to 6-year-olds assumed to be nonexposed by virtue of living in nonendemic country (*N* = 119). Because anti-TmpA antibodies may decrease after resolution of infection,^19,21^ the positive panel was restricted to samples that tested positive for TPPA and had a RPR titer of ≥ 4 (*N* = 15). The negative panel for TmpA consisted of 22 endemic-community samples that were TPPA-positive and RPR-negative, 47 that were both TPPA- and RPR-negative, and 74 samples from children from nonendemic areas (*N* = 143).

### Ethics.

The survey received nonresearch determination from CDC. It was reviewed and approved by the Bangladesh Institute of Epidemiology, Disease Control and Research institutional review board. All participation was voluntary, and the objectives, participation time, and risks and benefits of participation were explained to each participant prior to collecting data. The caregiver of each child provided verbal consent for participation. Verbal assent was obtained from older children.

### Data analysis.

Data were analyzed using GraphPad Prism v7.02 (La Jolla, CA) and R studio (Boston, MA). For analysis of treponemal-specific antibody responses, data were stratified into 1- to 4-year-olds, which likely reflect current yaws transmission status, and 5- to 14-year-olds (or 5- to 6-year-olds for Nayapara), the age range most likely to be affected by yaws. Analysis of *C. trachomatis*–specific antibody responses was restricted to 1- to 9-year-olds, the age range currently used for programmatic decision-making using clinical markers. Confidence intervals (CIs; 95%) were determined for nonzero data points; when antibody prevalence was 0, binomial exact calculation using one-sided 97.5% confidence intervals was used. CIs are not presented for data from Kutupalong, where sample collection was stopped early due to insecurity, because this truncated sampling introduces biases that prevent reliable estimation of uncertainty.

To estimate ocular *C. trachomatis* transmission intensity, we calculated the seroconversion rate (SCR) by fitting a simple reversible catalytic model to the measured seroprevalence, stratified into yearly age groups, using maximum likelihood methods.^22^ For these models, no seroreversion was assumed. SCRs were only estimated for *C. trachomatis* because seroprevalence to treponemal antigens is not anticipated to increase with age among 5- to 14-year-olds in endemic areas.

## RESULTS

**Figure 1.**
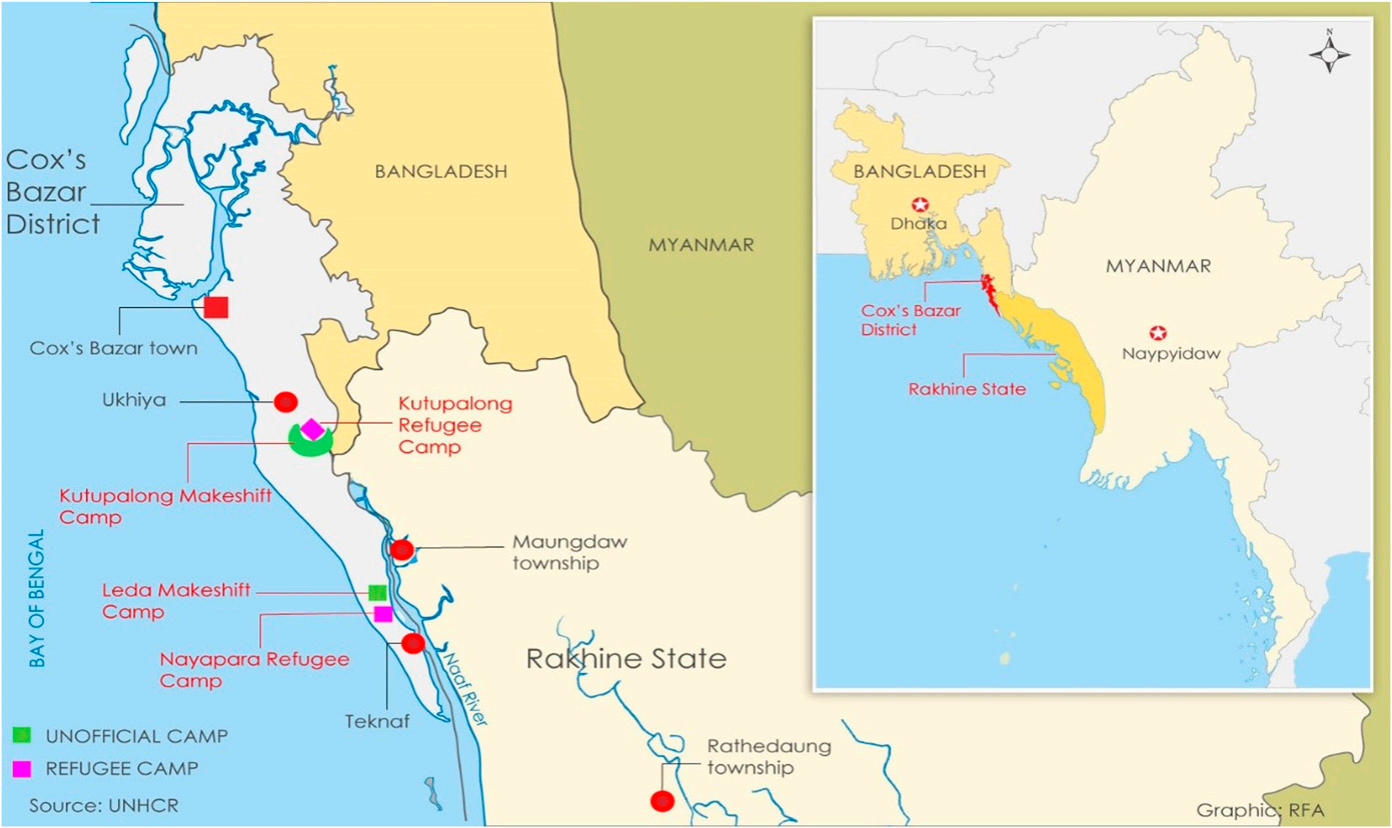
Location of sites in Cox’s Bazar, Bangladesh. The boundaries and names shown and the designations used on this map do not imply the expression of any opinion whatsoever on the part of the World Health Organization concerning the legal status of any country, territory, city, or area or of its authorities, or concerning the delimitation of its frontiers or boundaries. This figure appears in color at www.ajtmh.org.

Figure 1 shows the location of each of the camps included in the survey. Table 1 summarizes enrollment for Nayapara, Kutupalong, and the Makeshift Settlements. Adequate DBS samples were collected from 657 children in the Makeshift Settlements and 273 children in Nayapara.

**Table 1:** Enrollment and sex, by site

	Makeshift Settlements	Nayapara	Kutupalong
Number of households visited	715	524	410
Number enrolled	675 (94%)	483 (92%)	328 (80%)
Number not enrolled	40 (6%)	41 (8%)	82 (20%)
Not at home	39 (5%)	41 (8%)	20 (5%)
Refusal	1 (0.1%)	0 (0%)	62 (15%)
Number of children enrolled	829	638	394
Number eligible for DBS collection	789 (95%)	281 (44%)	390 (98.9%)
Number with adequate DBS collected	657 (83%)	273 (97%)	309 (79%)
Number without DBS collected	132 (17%)	8 (3%)	81 (21%)
Not at home	92 (12%)	6 (2%)	51 (13%)
Parent refused	3 (0.4%)	0 (0%)	14 (4%)
Child refused	15 (2%)	1 (0.4%)	14 (4%)
Insufficient blood draw	20 (3%)	1 (0.4%)	0 (0%)
Other reason (e.g., child visibly sick)	2 (0.3%)	0 (0%)	2 (1%)
Sex (female)	317 (44%)	126 (46%)	142 (46%)

DBS = dried blood spots. This table was adapted from Feldstein et al.^4^

### *C. trachomatis*–specific antibody responses.

In Nayapara, the prevalence of antibody responses among 1- to 6-year-olds (*N* = 273) was 1.5% (95% CI: 0.47–4.0) against Pgp3 and 7.0% (95% CI: 4.4–10.8) against CT694. In the Makeshift Settlements (*N* = 657), antibody responses among 1- to 9-year-olds was 1.4% (95% CI: 0.67–2.8) against Pgp3 and 2.8% (95% CI: 1.7–4.6) against CT694. The intensity of the antibody responses (in MFI-BG) by age is shown in Figure 3. The estimated SCR for Nayapara was 0.0043 per year (95% CI: 0.0032–0.0053) for Pgp3 and 0.029 per year (95% CI: 0.026–0.031) for CT694. For the Makeshift Settlements, SCR was estimated to be 0.0033 per year (95% CI: 0.0028–0.0038) for Pgp3 and 0.011 per year (95% CI: 0.0096–0.013) for CT694 (Figure 4).

### Treponemal-specific antibody responses.

**Figure 2.**
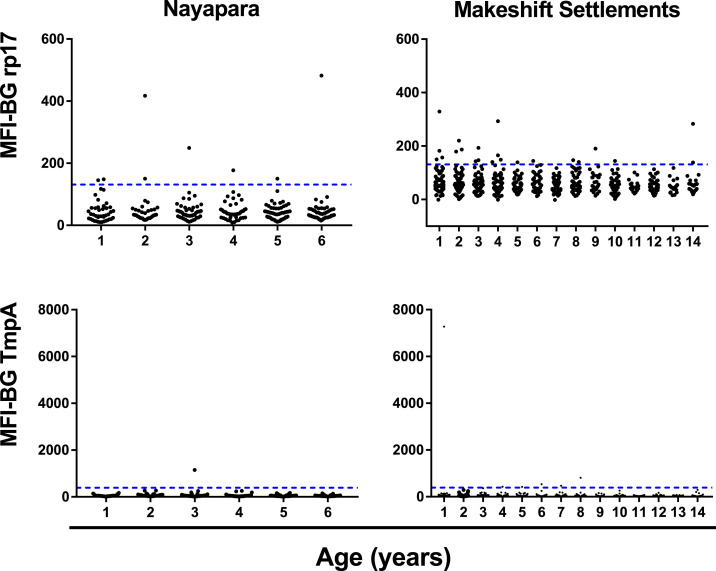
Intensity of antibody responses to the treponemal antigens rp17 (top) and TmpA (bottom) stratified by age in years up to 6 years for Nayapara Registered Camp (left) and up to 14 years for Makeshift Settlements (right). Horizontal lines indicate the cutoff for positivity. MFI-BG = median fluorescence intensity minus background. The upper range of the Y-axis scale differs for each antigen (600 for rp17 and 8,000 for TmpA, adjusted to be the same within each antigen to compare the two sites). TmpA = treponemal membrane protein A; rp17 = recombinant protein17. This figure appears in color at www.ajtmh.org.

In Nayapara, prevalence of antibody responses among 1- to 6-year-olds (*N* = 273) was 2.4% (95% CI: 0–5.0) against rp17, 0.37% (95% CI: 0–2.3) against TmpA, and 0% (97.5% CI: 0–1.7%) against both antigens (Table 2). In the Makeshift Settlements (*N* = 657), prevalence of antibody responses among 1- to 14-year-olds (*N* = 675) was 3.3% (95% CI: 2.1–5.0) against rp17, 0.91% (95% CI: 0.3–2.0) against TmpA, and 0.15% (95% CI: 0–0.8%) against both antigens. Table 2 shows the antibody data by site and stratified into 1- to 4-year-olds and 5- to 14-year-olds, the age group typically included in active yaws case identification. The intensity of the antibody responses (in MFI-BG) by age is shown in Figure 2.

**Table 2:** Antibody responses to treponemal antigens rp17 and TmpA by site

Site	Age (years)	Number sampled	Anti–rp17+		Anti–TmpA+		Anti–rp17 + and anti–TmpA+	
			*n*	% (95% CI)	*n*	% (95% CI)	*n*	% (95% CI)
Nayapara Registered Camp	1–4	147	5	3.4 (1.1–7.8)	1	0.68 (0–3.7)	0	0 (0–2.5)
	5–6	126	3	2.4 (0–5.0)	1	0.37 (0–2.3)	0	0 (0–17)
	All	273	8	2.9 (0.9–4.9)	2	0.73 (0–1.7)	0	0 (0–1.1)
Makeshift Settlements	1–4	246	13	5.3 (2.8–8.9)	2	0.81 (0.1–2.9)	1	0.4 (0–2.2)
	5–14	411	9	2.2 (1.0–4.1)	4	0.97 (0.3–2.5)	0	0 (0–0.9)
	All	657	22	3.3 (2.1–5.0)	6	0.91 (0.3–2.0)	1	0.15 (0–0.8)

CI = confidence interval; *n* = number positive; rp17 = recombinant protein17; TmpA = treponemal membrane protein A. When antibody prevalence was 0, binomial exact calculation using one-sided 97.5% CI was used.

### Data from abbreviated survey in Kutupalong.

Of 410 households visited before discontinuation of the survey in Kutupalong, 15% refused to participate and 5% were not at home. In the remaining 328 households, adequate DBS samples were collected from 309 children. No children were seropositive for antibodies to rp17 and only one child was seropositive for antibodies to TmpA. Three children were seropositive for antibodies to Pgp3 and 11 children were seropositive for antibodies to CT694. Table 1 shows enrollment information and demographic information from the households visited before cessation of the survey in Kutupalong. Supplemental Tables 1 and 2 show raw antibody data for treponemal and *C. trachomatis* antigens, respectively.

## DISCUSSION

Multiplex bead assays allow testing for antibodies against multiple antigens from a variety of pathogens in the same well, saving considerable laboratory processing and testing time and facilitating integrated surveillance efforts. This could be especially useful for surveillance of NTDs, which, as their “neglected” designation indicates, have significantly fewer resources devoted to their control than their importance as causes of human suffering would demand: NTDs are among the most common infections worldwide, affecting an estimated 2.9 billion people.^23^ In the current evaluation, testing for antibodies against the pathogens that cause trachoma and yaws was included as part of a larger serosurvey designed to measure vaccination coverage among children in communities in Cox’s Bazar, Bangladesh. This survey was itself embedded in a SMART (Standardized Monitoring and Assessment of Relief and Transition) nutritional survey, which seeks to assess the nutritional status of the under-5 population and mortality rate of the entire population in situations of humanitarian crisis.^24^ The data showed no serologic evidence for transmission of the pathogens causing yaws and trachoma in these communities.

The frequency of antibody responses to *C. trachomatis* antigens, especially Pgp3, in this population was exceptionally low, similar to that seen in U.S. children^25^ and children in districts previously endemic for trachoma in Nepal,^16^ the United Republic of Tanzania,^15^ and Ghana.^26^ In the three camps, the frequency of antibody responses was similar to or lower than the 6.2% (95% CI: 0.0–19.9%) threshold established in models used to predict trachoma as a public health problem.^13^ Where trachoma is a public health problem, the prevalence of anti–*C. trachomatis* antibodies typically increases with age in children. Models suggest SCRs < 0.015 per year (95% CI: 0.0–0.49) correlate with TF < 5%^13^; SCRs estimated for these camps was within that range or fell below that potential cutoff.

**Figure 3.**
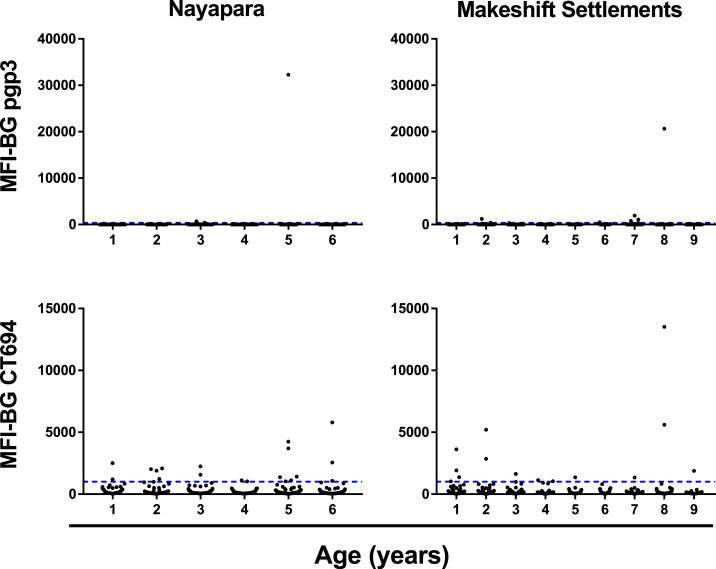
Intensity of antibody responses to the *Chlamydia trachomatis* antigens Pgp3 (top) and CT694 (bottom) stratified by age in years up to 6 years for Nayapara Registered Camp (left) and up to 9 years for Makeshift Settlements (right). Each dot represents an individual. Y-axis shows the intensity of the antibody response against each antigen measured in median fluorescence intensity (MFI) with background levels subtracted out (MFI-BG). The upper range of the Y-axis scale differs for each antigen (40,000 for Pgp3 and 15,000 for CT694, and then adjusted to be the same within each antigen to compare the two sites). The dashed blue line shows the seropositivity cutoff. CT = *C. trachomatis*; Pgp = plasmid gene product. This figure appears in color at www.ajtmh.org.

A potential limitation to interpreting these data is that in Nayapara, children aged 7 years and older were not included, potentially limiting our ability to detect possible increases in seroprevalence with age. Although having serological data on older children is ideal, community-based surveys in younger age groups^12^ in which TF prevalence was > 5% have shown an increase in seroprevalence by age. Therefore, based on the available data, an increase in seroprevalence by age should still have been observable in 1- to 6-year-olds in Nayapara if ocular *C. trachomatis* transmission was ongoing. The prevalence of antibody responses against CT694 was consistently higher than those against Pgp3, although these responses tend to cluster around the cutoff, as seen in Figure 3. Although the interpretation of serological data for trachoma should be made with caution because these tests are not currently included in WHO guidance for trachoma surveillance,^27^ the combination of very low seroprevalence to *C. trachomatis* antigens and low SCR, especially against Pgp3, is strongly suggestive of low or no transmission of ocular *C. trachomatis* in these populations.

**Figure 4.**
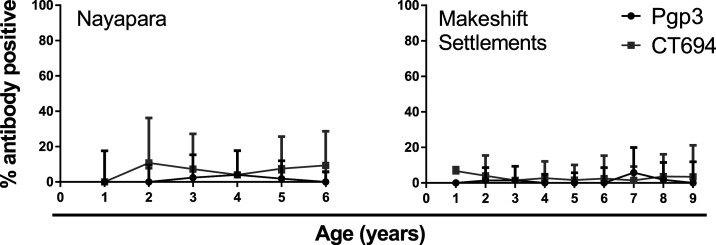
Seroprevalence of antibody responses to the *Chlamydia trachomatis* antigens Pgp3 (black line) and CT694 (gray line) by age. Vertical lines represent 95% confidence intervals. CT = *C. trachomatis*; Pgp = plasmid gene product.

The WHO has set an eradication target for yaws by 2020, but lack of data on where yaws is currently endemic and lack of funding for mapping necessitates creative approaches for determining where to prioritize surveys and interventions.^28^ One such approach is using integrated serological testing from samples collected from other surveys, as was done in this study. Use of recombinant antigens in an MBA is not part of the standard diagnostic criteria for yaws. However, TmpA has been shown to have high specificity for *T. pallidum*, and responses to TmpA decrease within a year of treatment^21^ and show good correlation with RPR titers.^19^ Rp17 is an immunodominant antigen from *T. pallidum*^29^ with a reactive concordance of 94% to 99% against traditional treponemal tests.^19^ Only one of the children in the three sites under evaluation tested positive for antibody to both rp17 and TmpA antigens, suggesting little or no yaws in these populations.

The early cessation of data collection in Kutupalong limits the representativeness of the seroprevalence estimates for this camp. The number of antibody-positive individuals enrolled before data collection interruption, although not conclusive, are suggestive that children in Kutupalong have also been relatively infrequently exposed to the pathogens causing yaws and trachoma.

One challenge of integrated surveillance is ensuring that the sampling strategy for the targeted disease is epidemiologically appropriate for evaluating nontargeted diseases. In this survey, the numbers of households and 1- to 9-year-olds included overall were similar to those included in standard baseline trachoma surveys.^30^ Therefore, although the sampling is likely adequate for trachoma, it is likely suboptimal for yaws, so care should be taken not to overinterpret the lack of children with antitreponemal antibodies as a complete lack of yaws in this setting. Guidance on the sampling strategy that yaws programs should adopt in baseline surveys has not yet been generated; the eradication target, which requires demonstration of absence of infection transmission, makes this particularly challenging. However, within the limits of available guidance and subject to the caveats identified here, these data suggest that neither yaws nor trachoma is likely to be affecting this population.

## Supplemental tables

Supplemental materials

## References

[1] Situation Report: Rohingya Refugee Crisis, InterSector Coordination Group. Cox’s Bazar. Available at: https://reliefweb.int/report/bangladesh/iscg-situation-report-rohingya-refugee-crisis-cox-s-bazar-july-2020.

[2] Summers A Humphreys A Leidman E Van Mil LT Wilkinson C Narayan A Mald Miah ML Cramer BG Bilukha O, 2018. Notes from the field: diarrhea and acute respiratory infection, oral cholera vaccination coverage, and care-seeking behaviors of Rohingya Refugees—Cox’s Bazar, Bangladesh, October–November 2017. MMWR Morb Mortal Wkly Rep 67: 533–535. 29746454 10.15585/mmwr.mm6718a6 PMC5944978

[3] World Health Organization, 2017. *Mortality and Morbidity Weekly Bulletin 8.* Geneva, Switzerland: WHO.

[4] Feldstein LR 2020. Vaccination coverage survey and seroprevalence among forcibly displaced Rohingya children, Cox’s Bazar, Bangladesh, 2018: a cross-sectional study. PLoS Med 17: e1003071. 32231368 10.1371/journal.pmed.1003071 PMC7108726

[5] World Health Organization, 1996. *Future Approaches to Trachoma Control: Report of a Global Scientific Meeting, Geneva, Switzerland, June 17–20, 1996*. Geneva, Switzerland: WHO.

[6] Solomon AWZM Kuper H Buchan JC Mabey DCW Foster A, 2006. *Trachoma Control: A Guide for Programme Managers*. Geneva, Switzerland: World Health Organization.

[7] World Health Organization. *Global Health Observatory Data Repository*. Geneva, Switzerland: WHO.

[8] Mitja O 2015. Global epidemiology of yaws: a systematic review. Lancet Glob Health 3: e324–e331. 26001576 10.1016/S2214-109X(15)00011-X PMC4696519

[9] Mitja O Hays R Ipai A Penias M Paru R Fagaho D de Lazzari E Bassat Q, 2012. Single-dose azithromycin versus benzathine benzylpenicillin for treatment of yaws in children in Papua New Guinea: an open-label, non-inferiority, randomised trial. Lancet 379: 342–347. 22240407 10.1016/S0140-6736(11)61624-3

[10] Cama A 2017. Prevalence of signs of trachoma, ocular *Chlamydia trachomatis* infection and antibodies to Pgp3 in residents of Kiritimati Island, Kiribati. PLoS Negl Trop Dis 11: e0005863. 28898240 10.1371/journal.pntd.0005863 PMC5609772

[11] Gwyn SE Xiang L Kandel RP Dean D Gambhir M Martin DL, 2018. Prevalence of *Chlamydia trachomatis*-specific antibodies before and after mass drug administration for trachoma in community-wide surveys of four communities in Nepal. Am J Trop Med Hyg 98: 216–220. 29141720 10.4269/ajtmh.17-0102 PMC5928690

[12] Martin DL 2015. Serological measures of trachoma transmission intensity. Sci Rep 5: 18532. 26687891 10.1038/srep18532 PMC4685243

[13] Pinsent A 2018. The utility of serology for elimination surveillance of trachoma. Nat Commun 9: 5444. 30575720 10.1038/s41467-018-07852-0 PMC6303365

[14] Migchelsen SJ 2017. Serology reflects a decline in the prevalence of trachoma in two regions of The Gambia. Sci Rep 7: 15040. 29118442 10.1038/s41598-017-15056-7 PMC5678181

[15] West SK Munoz B Weaver J Mrango Z Dize L Gaydos C Quinn TC Martin DL, 2016. Can we use antibodies to *Chlamydia trachomatis* as a surveillance tool for national trachoma control programs? Results from a district survey. PLoS Negl Trop Dis 10: e0004352. 26771906 10.1371/journal.pntd.0004352 PMC4714879

[16] West SK Zambrano AI Sharma S Mishra SK Munoz BE Dize L Crowley K Gaydos CA Rotondo LA, 2017. Surveillance surveys for reemergent trachoma in formerly endemic districts in Nepal from 2 to 10 years after mass drug administration cessation. JAMA Ophthalmol 135: 1141–1146. 28973295 10.1001/jamaophthalmol.2017.3062 PMC5710394

[17] Butcher R 2018. Clinical signs of trachoma are prevalent among Solomon Islanders who have no persistent markers of prior infection with *Chlamydia trachomatis*. Wellcome Open Res 3: 14. 29588922 10.12688/wellcomeopenres.13423.2 PMC5854984

[18] Cocks N 2016. Community seroprevalence survey for yaws and trachoma in the Western Division of Fiji. Trans R Soc Trop Med Hyg 110: 582–587. 27852877 10.1093/trstmh/trw069 PMC5155547

[19] Cooley GM 2016. Evaluation of multiplex-based antibody testing for use in large-scale surveillance for yaws: a comparative study. J Clin Microbiol 54: 1321–1325. 26962086 10.1128/JCM.02572-15 PMC4844712

[20] Gwyn S Cooley G Goodhew B Kohlhoff S Banniettis N Wiegand R Martin DL, 2017. Comparison of platforms for testing antibody responses against the *Chlamydia trachomatis* antigen Pgp3. Am J Trop Med Hyg 97: 1662–1668. 29016320 10.4269/ajtmh.17-0292 PMC5805053

[21] Ijsselmuiden OE Schouls LM Stolz E Aelbers GN Agterberg CM Top J van Embden JD, 1989. Sensitivity and specificity of an enzyme-linked immunosorbent assay using the recombinant DNA-derived *Treponema pallidum* protein TmpA for serodiagnosis of syphilis and the potential use of TmpA for assessing the effect of antibiotic therapy. J Clin Microbiol 27: 152–157. 2643617 10.1128/jcm.27.1.152-157.1989 PMC267251

[22] Drakeley CJ 2005. Estimating medium- and long-term trends in malaria transmission by using serological markers of malaria exposure. Proc Natl Acad Sci USA 102: 5108–5113. 15792998 10.1073/pnas.0408725102 PMC555970

[23] Hotez PJ Molyneux DH Fenwick A Kumaresan J Sachs SE Sachs JD Savioli L, 2007. Control of neglected tropical diseases. N Engl J Med 357: 1018–1027. 17804846 10.1056/NEJMra064142

[24] Action Against Hunger, 2018. Available at: https://actionagainsthunger.ca/program-areas/smart/. Accessed July 25, 2018.

[25] Goodhew EB 2012. CT694 and pgp3 as serological tools for monitoring trachoma programs. PLoS Negl Trop Dis 6: e1873. 23133684 10.1371/journal.pntd.0001873 PMC3486877

[26] Senyonjo LG 2018. Serological and PCR-based markers of ocular *Chlamydia trachomatis* transmission in northern Ghana after elimination of trachoma as a public health problem. PLoS Negl Trop Dis 12: e0007027. 30550537 10.1371/journal.pntd.0007027 PMC6310292

[27] World Health Organization, 2017. *Trachoma Alternative Indicators Study Review: 31 August–1 September 2016*. Geneva, Switzerland: WHO.

[28] Fitzpatrick C Asiedu K Solomon AW Mitja O Marks M Van der Stuyft P Meheus F, 2018. Prioritizing surveillance activities for certification of yaws eradication based on a review and model of historical case reporting. PLoS Negl Trop Dis 12: e0006953. 30513075 10.1371/journal.pntd.0006953 PMC6294396

[29] Rostopira N Tkacikova L Rayevska G Pylypenko V Mikula I Spivak M, 2003. Elaboration of enzyme immunoassay based on recombinant antigens and intended for diagnostics of syphilis. Folia Microbiol (Praha) 48: 549–553. 14533489 10.1007/BF02931339

[30] Solomon AW 2015. The Global Trachoma Mapping Project: methodology of a 34-country population-based study. Ophthalmic Epidemiol 22: 214–225. 26158580 10.3109/09286586.2015.1037401 PMC4687001

