# Supplementary material for: No Serological Evidence of Trachoma or Yaws Among Residents of Registered Camps and Makeshift Settlements in Cox’s Bazar, Bangladesh
Source: Am J Trop Med Hyg. 2021 May 3;104(6):2031–7. doi: 10.4269/ajtmh.21-0124 (PMC8176462; doi:10.4269/ajtmh.21-0124)
Supplement: Supplementary file 1 [file tpmd210124.SD1.pdf]

Supplemental Tables – Kutupalong Camp

Supplemental Table 1 – antibody positivity to treponemal antigens stratified into two age groups in the Kutupalong Camp.

| Age (years) | N | rp17+<br>n (%) | TmpA+<br>n (%) | rp17+ TmpA+<br>n (%) |
|---|---|---|---|---|
| 1–4 | 86 | 0 (0) | 1 (1·2) | 0 (0) |
| 5–14 | 223 | 0 (0) | 0 (0) | 0 (0) |
| All | 309 | 0 (0) | 1 (0·3) | 0 (0) |

Supplemental Table 2 – antibody positivity to *C. trachomatis* antigens by year of age in the Kutupalong Camp.

| | | **Pgp3** | **CT694** |
|---|---|---|---|
| **Age (years)** | **N** | **n** | **N** |
| 1 | 7 | 0 (0) | 0 (0) |
| 2 | 26 | 0 (0) | 0 (0) |
| 3 | 33 | 0 (0) | 2 (6·1) |
| 4 | 20 | 0 (0) | 0 (0) |
| 5 | 36 | 1 (2·8) | 4 (11·1) |
| 6 | 34 | 0 (0) | 0 (0) |
| 7 | 26 | 0 (0) | 2 (7·7) |
| 8 | 23 | 1 (4·3) | 2 (8·7) |
| 9 | 17 | 1 (5·9) | 1 (5·9) |
| All ages | 222 | 3 (1·4) | 11 (4·9) |

Pgp = plasmid gene product. CT = *C. trachomatis*; TmpA = treponemal membrane protein A; rp17 = recombinant protein17
